# Baicalin Inhibits IL-17-Mediated Joint Inflammation in Murine Adjuvant-Induced Arthritis

**DOI:** 10.1155/2013/268065

**Published:** 2013-06-12

**Authors:** Xue Yang, Ji Yang, Hejian Zou

**Affiliations:** ^1^Division of Rheumatology, Huashan Hospital, Fudan University, 12 Wulumuqizhong Road, Shanghai 200040, China; ^2^Institute of Rheumatology, Immunology and Allergy, Shanghai Medical College, Fudan University, Huashan Hospital, Shanghai 200040, China; ^3^Department of Dermatology, Zhongshan Hospital, Fudan University, Shanghai 200032, China

## Abstract

T-helper-17 (Th17) cells are implicated in a number of inflammatory disorders including rheumatoid arthritis. Antagonism of Th17 cells is a treatment option for arthritis. Here, we report that Baicalin, a compound isolated from the Chinese herb Huangqin (*Scutellaria baicalensis* Georgi), relieved ankle swelling and protected the joint against inflammatory destruction in a murine adjuvant-induced arthritis model. Baicalin inhibited splenic Th17 cell population expansion *in vivo*. Baicalin prevented interleukin- (IL-) 17-mediated lymphocyte adhesion to cultured synoviocytes. Baicalin also blocked IL-17-induced intercellular adhesion molecule 1, vascular cell adhesion molecule 1, IL-6, and tumor necrosis factor-alpha mRNA expression in cultured synoviocytes. Collectively, these findings suggest that Baicalin downregulates the joint inflammation caused by IL-17, which is likely produced by an expanded population of splenic Th17 cells in experimental arthritis. Baicalin might be a promising novel therapeutic agent for treating rheumatoid arthritis in humans.

## 1. Introduction

Rheumatoid arthritis (RA) is an autoimmune disease characterized by chronic inflammation of the joint synovium, ultimately leading to cartilage and bone erosion and joint destruction [1]. T and B lymphocytes play pivotal roles in RA initiation and propagation [1]. T lymphocytes activate a wide range of effector cells in the synovium by direct cell-cell contact and by releasing inflammatory cytokines. These activated effector cells also release a spectrum of proinflammatory mediators including tumor necrosis factor alpha (TNF*α*), interleukin- (IL)-1, IL-6, and IL-17, which are responsible for joint inflammation and destruction [2, 3]. More recently, IL-17 has been suggested to play important additional key roles in RA induction and maintenance [4–6]. 

Interleukin 17 is elevated in synovial fluids of RA patients and in inflamed joints of experimental arthritis mouse models [4, 5, 7]. IL-17 is a proinflammatory cytokine produced predominantly by T-helper-17 (Th17) cells [8, 9]. IL-17 is an important regulator of immune and inflammatory responses, including the induction of other proinflammatory cytokines and osteoclastic bone resorption [6, 10]. This cytokine is found in synovial fluids and synoviocytes of RA patients and is produced by both Th17 cells and RA synoviocytes [2, 6]. IL-17 induces inflammatory cell infiltration into the synovium, enhances collagen degradation and decreases collagen synthesis in cartilage, reduces proteoglycan synthesis by chondrocytes, and increases bone resorption and erosion [11, 12]. Thus, inhibiting IL-17 might provide important therapeutic benefits in halting the joint destruction that occurs in RA.

Baicalin (7-glucuronic acid, 5,6-dihydroxyflavone; molecular weight = 446.36) is a flavonoid compound originally isolated from the root of the Chinese herb Huangqin (*Scutellaria baicalensis* Georgi). Baicalin has a clinically proven safety record and is used as an anti-inflammatory drug in traditional Chinese medicine [13, 14]. Our previous studies showed that Baicalin inhibits Th17 cell differentiation *in vitro* and *in vivo* and that Baicalin administration relieves lupus nephritis symptoms by inhibiting Th17 cell infiltration into the kidney. Baicalin also has an apparent antagonistic role on Th17 cell-mediated inflammation in lupus-prone MRL/lpr mice [15]. However, the possible biological effects and mechanisms of action of Baicalin in RA models remain unexplored.

In the current study, we demonstrated that Baicalin relieved ankle swelling and protected the ankle joint against inflammatory destruction in a murine adjuvant-induced arthritis model. Baicalin inhibited splenic Th17 cell population expansion *in vivo*, prevented IL-17-mediated lymphocyte adhesion to synoviocytes, and inhibited IL-17-mediated adhesion molecule expression and cytokine expression by synoviocytes. Collectively, these findings demonstrate that Baicalin decreases splenic Th17 cell population expansion and inhibits IL-17-mediated inflammatory joint injury in experimental arthritis. Thus, Baicalin may be a promising therapeutic agent for treating RA in humans.

## 2. Materials and Methods

### 2.1. Mice, Adjuvant-Induced Arthritis, and Baicalin Treatment

 Male C57BL/6 (B6) mice were purchased from the Shanghai Laboratory Animal Center (Chinese Academy of Sciences). Animal protocols were approved by the Institutional Animal Care and Use Committee of Zhongshan Hospital, Fudan University, Shanghai, China. Mice were maintained under pathogen-free conditions. Adjuvant arthritis was induced using a modification of previously published methods [16, 17]. Briefly, 6- to 8-week-old male B6 mice were immunized with 100 *μ*L of heat-inactivated *Mycobacterium tuberculosis* (10 mg/mL) that was emulsified in complete Freund's adjuvant (both from Sigma-Aldrich, St. Louis, MO, USA). Mice were injected subcutaneously in the right hind footpad on day 0. Baicalin (purity > 98%; National Institute for the Control of Pharmaceutical and Biological Products, Beijing, China) was dissolved in phosphate-buffered saline (PBS) prior to experimentation. Either 100 *μ*L Baicalin solution (100 mg/kg) or an equivalent volume of PBS (vehicle control) was injected intraperitoneally into mice on a daily basis between postimmunization days 14 to 21. To quantify joint swelling, ankle widths were measured with a micrometer.

### 2.2. Histopathology

At the time of sacrifice (21 days after immunization), the hind paws were fixed in buffered formalin. After decalcification in 10% formic acid for 48 hours, the tissues were processed for paraffin embedding, and ankle joint sections were stained with hematoxylin and eosin. The slides were interpreted by a researcher who was blinded to the animal treatment group. A minimum of five ankle sections per mouse were assessed and scored for a semiquantitative measure of subsynovial inflammation, synovial hyperplasia, pannus formation, cartilage erosion, and bone destruction. The scoring ranged from 0 to 2 for each parameter, with 0 indicating no observed pathology and 2 indicating maximal pathological changes, as previously detailed [18]. Overall pathology scores were the sum of the values for the individual criteria. Spleens were collected at sacrifice to calculate the spleen size index (spleen weight (g) divided by total body weight (g)).

### 2.3. Cytokine Staining and Flow Cytometry Analysis

For Th17 cell detection, spleen cells from mice were activated for 5 hours with 50 ng/mL phorbol myristate acetate and 750 ng/mL ionomycin in the presence of 20 *μ*g/mL brefeldin A (all from Sigma-Aldrich) in a tissue culture incubator at 37°C. Surface staining with FITC-conjugated anti-CD4 antibody (eBioscience, San Diego, CA, USA) was performed for 15 min in the dark, and cells were resuspended in fixation/permeabilization solution according to the manufacturer's instructions (Invitrogen, Carlsbad, CA, USA). Negative controls were stained with FITC-conjugated isotype-matched antibody. Intracellular IL-17 staining was performed with phycoerythrin-conjugated anti-IL-17 antibody, with phycoerythrin-conjugated isotype-matched antibody serving as the control. Cell staining was performed according to the manufacturer's protocol (eBioscience). We first gated on CD4^+^ T cells, and then dual-positive CD4^+^IL-17^+^ cells were assessed in a CD4^+^ gate, using a FACS-Calibur flow cytometer (BD Biosciences, San Jose, CA, USA). Flow cytometry data were further analyzed with FlowJo software (Tree Star, San Carlos, CA, USA).

### 2.4. Synoviocyte Culture, Cytokine Stimulation, and T-Cell Coculture

Synoviocytes were isolated as previously described [19]. Synoviocytes were seeded into 12-well tissue culture plates (10,000 cells/well) in Dulbecco's modified Eagle's medium (DMEM; Hyclone, Logan, UT, USA) and allowed to adhere for 24 hours. Synoviocyte monolayers were stimulated with 50 ng/mL IL-17 (eBioscience) for 24 hours with or without inclusion of 20 *μ*M Baicalin. After synoviocyte stimulation, 50,000 splenocytes isolated from B6 mice were added to the synoviocyte cultures in fresh DMEM, and the coculture was performed for an additional 24 hours. Synoviocyte cultures were washed twice to remove nonadherent splenocytes. Splenocyte adhesion to synoviocytes was determined by counting splenocytes in five random fields/well under 200x magnification.

### 2.5. RNA Isolation and Real-Time Reverse Transcription-Polymerase Chain Reaction (RT-PCR)

The mRNA expression of intercellular adhesion molecule-1 (ICAM-1), vascular cell adhesion molecule-1 (VCAM-1), IL-6, and TNF-*α* in spleen, joint tissue, and IL-17-simulated synoviocytes was analyzed by real-time RT-PCR. Total RNA was purified with Trizol reagent (Invitrogen), cDNAs were synthesized using the Primescript RT Master Mix Perfect Real-Time Kit (TaKaRa, Tokyo, Japan), and mRNA expression levels were examined by cDNA amplification using a Bio-Rad iCycler 7500 Optical System (Bio-Rad, Richmond, CA, USA) using a SYBR Premix EX Taq Real-time PCR Master Mix (TaKaRa). The 2^−ΔΔCt^ method was used to normalize target gene transcription to *β*-actin expression (internal control) to calculate fold induction of target mRNA. The primer pairs were used are shown in Table 1. 

### 2.6. Statistical Analysis

Data are expressed as mean ± standard deviation for the appropriate units. Statistical significance was determined by analysis of variance followed by a Bonferroni *post hoc* test for multiple comparisons, or by Student's *t*-test. All *P* values ≤ 0.05 were considered indicative of statistically significant differences between test groups.

## 3. Results

### 3.1. Baicalin Alleviates Inflammatory Joint Injury in Murine Adjuvant-Induced Arthritis

**Figure 1:**
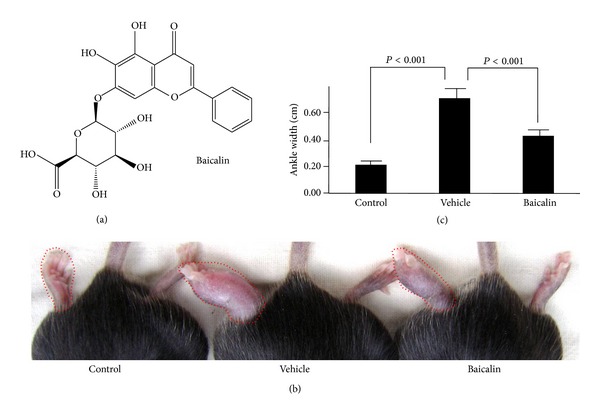
Baicalin alleviates ankle inflammation in a murine adjuvant-induced arthritis model. (a) Chemical structure of Baicalin. (b) Adjuvant-induced arthritis mice were injected intraperitoneally daily with 100 mg/kg Baicalin or phosphate-buffered saline (vehicle control) between postimmunization days 14 to 21 and photographs of the swollen ankles were acquired. (c) Ankle widths of the adjuvant-induced arthritis mice (*n* = 6 for each group).

**Figure 2:**
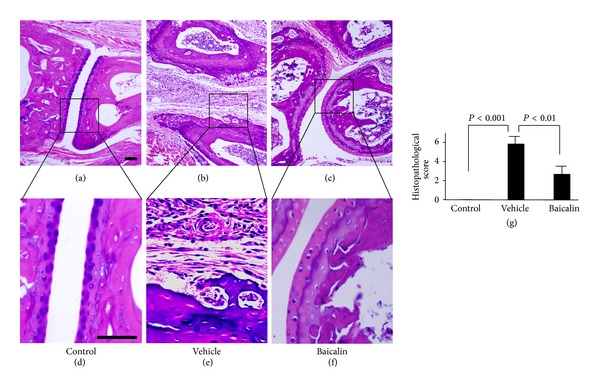
Baicalin inhibits ankle joint inflammation injury in adjuvant-induced arthritis mice. Adjuvant-induced arthritis mice were injected intraperitoneally daily with 100 mg/kg Baicalin or phosphate-buffered saline (vehicle control) for 1 week. Hematoxylin and eosin staining of control mice (a), adjuvant-induced arthritis mice with vehicle control treatment (b), and adjuvant-induced arthritis mice with Baicalin treatment (c). Further magnification of the black-bordered box showed the typical inflammatory injuries in control mice (d), adjuvant-induced arthritis mice with vehicle control treatment (e), and adjuvant-induced arthritis mice with Baicalin treatment (f). (g) Histopathological scores of the joint tissues (*n* = 6 for each group).

To determine whether Baicalin affects joint inflammation injury in murine adjuvant-induced arthritis, male B6 mice were immunized in the footpad with *Mycobacterium tuberculosis* emulsified in complete Freund's adjuvant on Day 0. Baicalin (100 mg/kg, Figure 1(a)) or PBS vehicle were injected intraperitoneally daily from day 14 to 21. Adjuvant/*Mycobacterium* injection caused severe ankle swelling in the mice, and Baicalin treatment clearly inhibited ankle inflammation (Figures 1(b) and 1(c)) and reduced joint injury. Histological evaluation showed that Baicalin inhibited inflammatory cell infiltration, synovial hyperplasia, and cartilage and bone erosion in arthritic mouse ankles (Figures 2(a)–2(f)). Finally, overall pathological assessment derived showed that Baicalin treatment significantly improved joint injury scores in arthritic mouse ankles (Figure 2(g)). These data demonstrate that Baicalin relieved inflammatory joint destruction in the adjuvant-induced arthritis mouse.

### 3.2. Baicalin Inhibits Splenic Th17 Cell Expansion *In Vivo*

**Figure 3:**
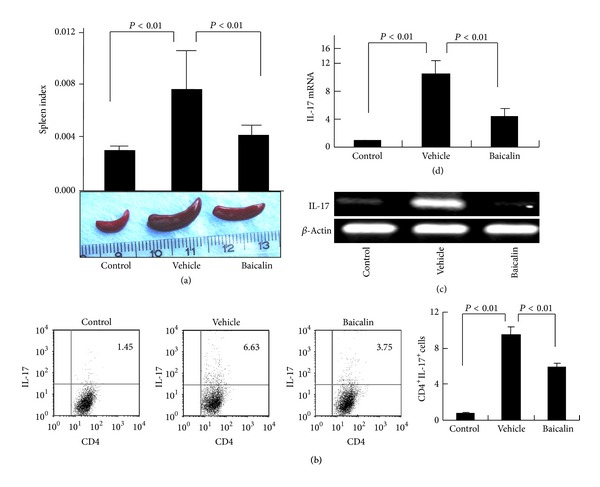
Baicalin inhibits splenic Th17 cell expansion *in vivo*. Adjuvant-induced arthritis mice were injected intraperitoneally daily with 100 mg/kg Baicalin or phosphate-buffered saline (vehicle control) between postimmunization days 14 to 21. (a) Spleen index of control and adjuvant-induced arthritis mice with or without Baicalin treatment (*n* = 6 for each group). Spleen photographs are shown in the lower panels (b). Isolated splenocytes were stained and sorted by flow cytometry first for CD4^+^ T cells. IL-17^+^ T cells were analyzed in the CD4^+^ gate (left). The percentage of dual positive CD4^+^IL-17^+^ cells among the gated CD4^+^ T cell fraction in each treatment group is shown on the right (*n* = 6 for each group). (c) IL-17 gene expression in joint tissues was analyzed by RT-PCR. These experiments were performed three times with similar results. (d) IL-17 gene expression in joint tissues was analyzed by real-time RT-PCR (*n* = 6 for each group).

Th17 cell-derived IL-17 is a potent inflammatory cytokine that mediates leukocyte infiltration and tissue destruction [9, 20]. Baicalin treatment for 1 week significantly inhibited splenic enlargement and reduced the spleen index in adjuvant-induced arthritis mice (Figure 3(a)). The percentage of the CD4^+^IL-17^+^ Th17 splenocyte population was significantly expanded in adjuvant-induced arthritis mice (9.47 ± 0.86% of total splenocytes, *n* = 6) compared to control mice (0.82 ± 0.11%, *n* = 6, *P* < 0.01; Figure 3(b)), and Baicalin treatment significantly inhibited splenic Th17 cell population expansion by nearly 40% (5.9 ± 0.47%, *n* = 6). ROR*γ*t, which is a key transcription factor involved in Th17 cell differentiation [21], was upregulated in spleen of adjuvant-induced arthritis mice, and Baicalin treatment significantly inhibited ROR*γ*t gene expression (Figure S1a in the Supplementary Material available online at http://dx.doi.org/10.1155/2013/268065). IL-17 mRNA expression was increased in the ankle joint tissues of adjuvant-induced arthritis mice compared to control mice, and Baicalin treatment significantly inhibited this upregulated IL-17 mRNA expression (Figures 3(c) and 3(d)). Although CD4^+^Foxp3^+^ Treg cells were expanded in spleen of adjuvant-induced arthritis mice, Baicalin did not significantly affect the frequency of Treg cells *in vivo *(Figures S1b and S1c). These data suggest that splenic Th17 cell expansion occurs in adjuvant-induced arthritis mice, and that Baicalin administration inhibits this Th17 cell expansion *in vivo*.

### 3.3. Baicalin Inhibits IL-17-Mediated Inflammation in Synoviocytes

**Figure 4:**
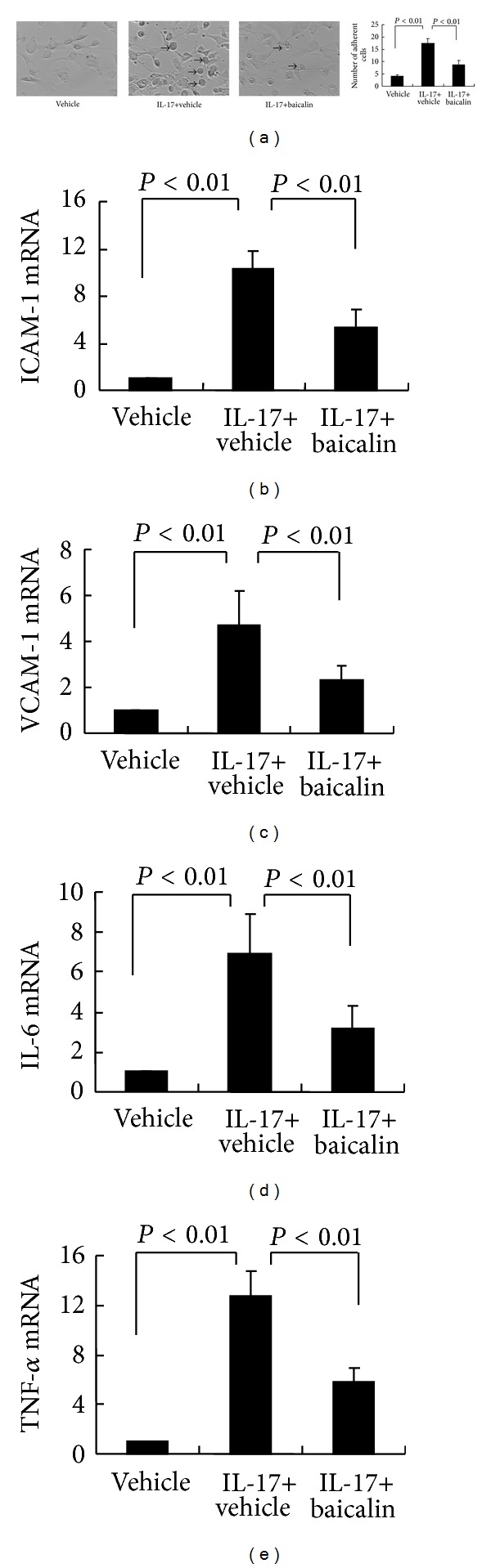
Baicalin inhibits IL-17-mediated inflammation reactions *in vitro*. (a) Synoviocytes (10,000 cells/well) were plated in 12-well tissue culture plates, allowed to adhere, and were then stimulated with 50 ng/mL IL-17 with or without 20 *μ*M Baicalin for 24 hours. Following IL-17/Baicalin stimulation, isolated splenocytes (50,000 cells/well) were added to the synoviocyte monolayers for another 24 hours. After washing twice, splenocyte adherence to synoviocytes was enumerated by microscopy at 200x magnification. Arrows indicate adhesive splenocytes (left). The number of adherent splenocytes is shown on the right. These experiments were performed three times with similar results. In mRNA expression studies, synoviocytes were cultured with 50 ng/mL IL-17 in the presence or absence 20 *μ*M Baicalin for 24 hours, and gene expression levels of ICAM-1 (b), VCAM-1 (c), IL-6 (d), and TNF-*α* (e) were examined by real-time RT-PCR. These experiments were performed three times with similar results.

Inflammatory cell infiltration into joint tissue mediates tissue chronic inflammation and tissue destruction in RA. IL-17 is thought to be a key cytokine that initiates inflammatory cell infiltration and subsequent joint injury in RA [11, 12]. To test this hypothesis, synoviocytes were isolated and stimulated with IL-17 and were subsequently cocultured with isolated splenocytes. The data showed that splenocyte adhesion to synoviocytes was upregulated when synoviocytes were prestimulated with IL-17-stimulated synoviocytes (Figure 4(a)), and inclusion of Baicalin during the adhesion assay significantly reduced splenocyte adhesion (Figure 4(a)). IL-17 increases the expression of genes encoding adhesion molecules and inflammatory cytokines [10]. We showed that stimulation of cultured synoviocytes with IL-17 for 24 hours upregulated the gene expression of the adhesion molecules ICAM-1 and VCAM-1 and induced gene transcription of the inflammatory cytokines IL-6 and TNF-*α*  (Figures 4(b)–4(e)). Interestingly, treatment with Baicalin significantly blocked the gene expression of these inflammatory mediators. These data suggest that IL-17 mediates inflammatory cell adhesion to synoviocytes and perpetuates the inflammatory cascade in the arthritic joint and that Baicalin blocks this IL-17-induced inflammatory cascade.

## 4. Discussion

Conventional disease-modifying antirheumatic drugs, nonsteroidal anti-inflammatory drugs, glucocorticosteroids, and the newer generation of biological agents comprise current drug therapies for RA. However, these drugs have limited efficacy, delayed onset of action, and long-term side effects and toxicity. Therapy using biological agents, such as TNF-*α* and IL-1 antagonists, despite substantial efficacy, entails high cost and may result in detrimental effects such as tumors and serious infections [22]. More effective, safe, and affordable drugs are needed to control RA. Many natural compounds exhibit anti-inflammatory properties and have potential for treating inflammatory disorders such as RA [3, 22]. 

Baicalin has a clinically proven safety record and has been used as an anti-inflammatory drug in traditional Chinese medicine [13]. Baicalin is extensively used in China to treat hepatitis. Baicalin possesses anti-inflammatory, antioxidant, and antiallergic properties and appears to alleviate symptoms of several chronic inflammatory diseases, including hepatitis, allergic diseases, and experimental autoimmune encephalomyelitis [14, 23–25]. Previous studies showed that Baicalin inhibits mononuclear cell proliferation, inhibits macrophage activation, and reduces the production of Th1 and Th17 cell-related cytokines in different murine disease models [15, 24, 26]. In the current study, we demonstrate that Baicalin effectively inhibits ankle swelling in an *in vivo* experimental arthritis model and reduces joint inflammation injury and destruction. This implies that Baicalin could be a promising agent for treating RA in humans. 

RA is characterized by aseptic joint inflammation in concert with upregulated inflammatory cytokines by the synovium and infiltrating leukocytes [1]. Increased inflammatory cytokine expression in the RA joint enhances and sustains inflammatory processes that cause joint tissue damage. Cytokine inhibition suppresses these inflammatory cascades and provides improved functional recovery in experimental models [1, 27]. In our study, the Th17 cell population was expanded in the spleens of adjuvant-induced arthritis mice. IL-17 gene expression was upregulated in arthritic joints tissues. It is possible that Th17 cell-derived IL-17 plays a key role in the pathogenesis of adjuvant-induced arthritis. Th17 cell-derived IL-17 could initiate or aggravate inflammation by inducing the production of cytokines, adhesion molecules, and other inflammatory molecules in different cell types within the joint [10, 27–29]. We previously showed that inhibiting Th17 cell expansion ameliorates joint injury in a murine collagen-induced arthritis model [30]. In the present study, we found that IL-17 induced the expression of adhesion molecules such as ICAM-1 and VCAM-1, which mediate lymphocyte adhesion to synoviocytes [10, 29]. IL-17 also elicited synovial expression of the cytokines IL-6 and TNF-*α*, which are known mediators of synovial inflammation in RA. These data suggest that IL-17 might be an upstream cytokine in RA pathogenesis, and the immune crosstalk between cytokines or cytokines/adhesion molecules might play an important role during the course of arthritis in the AA model [17]. We further showed that Baicalin effectively inhibits the expansion of splenic Th17 cells in adjuvant-induced arthritis mice. Baicalin inhibits the IL-17-upregulated expression of synovial adhesion molecules and inflammatory cytokines and subsequently prevents lymphocytes from adhering to synoviocytes. Inflammatory cytokine like IL-6 is a key cytokine for the development of Th17 cells [10], and our previous data and other studies showed that Baicalin treatment could inhibit IL-6 production and block IL-6-IL-6 receptor mediated ROR*γ*t transcription, which resulted in the inhibition of Th17 cell differentiation [15, 31, 32]. All together, these data indicated that Baicalin might prevent the perpetuation of the malignant inflammatory cascade in RA. Although our data prove that Baicalin inhibits IL-17-mediated inflammatory reactions in an experimental mouse arthritis model, further work is required to deepen our understanding of the underlying biological mechanisms.

Our data showed that Treg cells were also expanded in adjuvant-induced arthritis mouse, which was consistent with previous results [33]. Immunological homeostasis exemplifies the capacity of the immune system to upregulate immunosuppressive responses, which may ultimately limit the deterioration caused by autoimmunity [34]. The upregulation of Treg cells may reflect a feedback regulatory mechanism that is activated to minimize harmful autoimmune responses. Our previous observation indicated that Baicalin can enhance Foxp3 expression *in vitro* [25], whereas Baicalin treatment in adjuvant-induced arthritis mouse only slightly affected CD4^+^Foxp3^+^ T cells. This minor affection of Foxp3^+^ Treg cells *in vivo* might stem from the strong and complicated interference of excessive inflammatory cytokines network in adjuvant-induced arthritis mouse. 

In conclusion, we showed that splenic Th17 cell expansion occurs in murine adjuvant-induced arthritis, and IL-17-mediated inflammatory reactions play a key role in joint disease development in this model. Baicalin inhibits splenic Th17 cell population expansion *in vivo*, which prevents expansion of the IL-17-mediated inflammatory cascade and effectively reduces joint inflammatory injury in experimental arthritis. Taken together, these findings suggest that Baicalin might be a promising therapeutic agent for treating RA in humans.

## Supplementary Material

Figure S1: Baicalin regulate ROR*γ*t and Foxp3 expression.

## Figures and Tables

**Table 1:** 

Gene	Forward (5′-3′)	Reverse (5′-3′)
Mus *β*-actin	GACGGCCAGGTCATCACTATTG	AGGAAGGCTGGAAAAGAGCC
Mus IL-17	GGGAGAGCTTCATCTGTGTCTC	GGTTGACCTTCACATTCTGGA
Mus ICAM-1	CAATTTCTCATGCCGCACAG	AGCTGGAAGATCGAAAGTCCG
Mus VCAM-1	TGAACCCAAACAGAGGCAGAGT	GGTATCCCATCACTTGAGCAGG
Mus IL-6	GAGGATACCACTCCCAACAGACC	AAGTGCATCATCGTTGTTCATACA
Mus TNF*α*	TCTTCTCATTCCTGCTTGTGG	CACTTGGTGGTTTGCTACGAC
