# Supplementary material for: Baicalin Inhibits IL-17-Mediated Joint Inflammation in Murine Adjuvant-Induced Arthritis
Source: Clin Dev Immunol. 2013 Jun 12;2013:268065. doi: 10.1155/2013/268065 (PMC3694363; doi:10.1155/2013/268065)
Supplement: Supplementary file 1 — Figure S1: Baicalin regulate RORγt and Foxp3 expression. [file 268065.f1.doc]

**Supplemental Figure Legends**

**Figure S1.** **Baicalin regulate RORγt and Foxp3 expression.** Adjuvant-induced arthritis mice were injected intraperitoneally daily with 100 mg/kg Baicalin or phosphate-buffered saline (vehicle control) between post-immunization days 14 to 21. **(A)** RORγt gene expression in isolated splenocytes was analyzed by real time RT-PCR (n=6 for each group). **(B)** Isolated splenocytes were stained. Foxp3^+^ T cells were analyzed in the CD4^+^ gate according to the manufacturer’s instructions. **(C)** The percentage of dual positive CD4^+^Foxp3^+^ cells among the gated CD4^+^ T cell fraction in each treatment group are shown t (n=6 for each group).

**Figure S1**

**
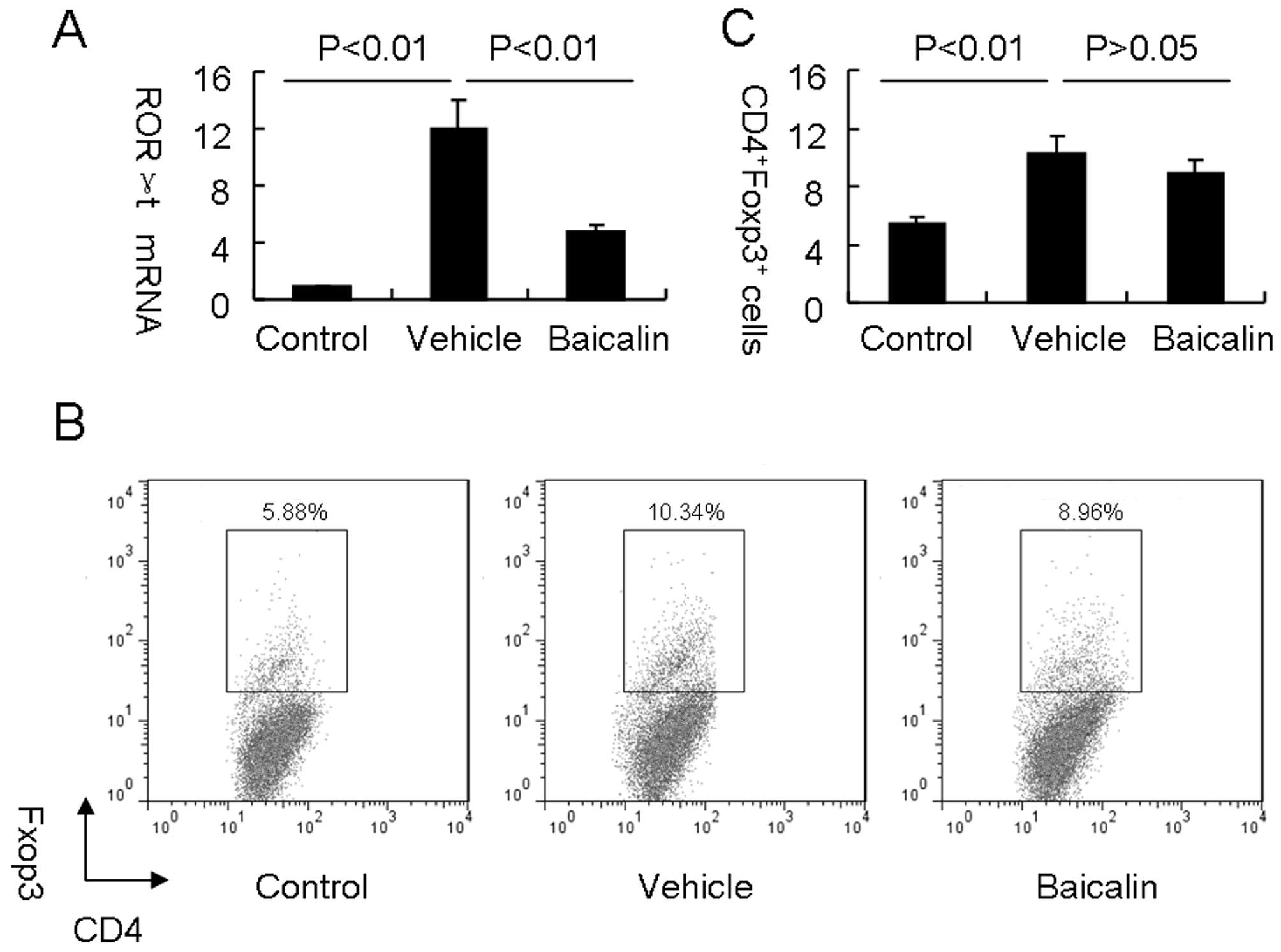
**
